# Effects of particulate air pollution exposure on lung-brain axis and related miRNAs modulation in mouse models

**DOI:** 10.3389/fcell.2025.1526424

**Published:** 2025-03-20

**Authors:** Alessandro Giammona, Giulia Terribile, Paolo Rainone, Chiara Pellizzer, Danilo Porro, Antonio Cerasa, Giulio Sancini, Ameen-Ur Rashid, Sara Belloli, Silvia Valtorta, Alessia Lo Dico, Gloria Bertoli

**Affiliations:** ^1^ Istituto di Bioimmagini e Sistemi Biologici Complessi (IBSBC), National Research Council (CNR), Segrate, Italy; ^2^ National Biodiversity Future Center (NBFC), Palermo, Italy; ^3^ Human Physiology Unit, School of Medicine and Surgery, University of Milano-Bicocca, Monza, Italy; ^4^ Department of Earth and Environmental Sciences, POLARIS Research Centre, University of Milano-Bicocca, Milano, Italy; ^5^ NeuroMI - Milan Centre for Neuroscience, University of Milano-Bicocca, Milano, Italy; ^6^ PhD Program, Program in Neuroscience, Medicine and Surgery Department, University of Milano-Bicocca, Milano, Italy

**Keywords:** air pollution, miRNA, mice, lung-brain axis, cancer and imaging

## Abstract

Particulate matter exposure is linked to numerous health issues, including respiratory, cardiovascular, and neurodegenerative diseases. This review focuses on the biological mechanisms through which air pollution influences the lung-brain axis, highlighting the role of miRNAs in regulating gene pathways affected by PM. Some microRNAs (miRNAs) are identified as key modulators of cellular processes, including inflammation, epithelial-to-mesenchymal transition (EMT), and blood-brain barrier integrity. Using mice models to study these effects allows for controlled experimentation on the systemic distribution of PM across biological barriers. Among the imaging technologies, Positron Emission Tomography is the best approach to monitor the distribution and effects of PM *in vivo*. The research underscores the importance of miRNA profiles as potential markers for the health effects of PM exposure, suggesting that specific miRNAs could serve as early indicators of damage to the lung-brain axis.

## 1 Introduction

World Health Organization (WHO) states that air pollution refers to any harmful chemical, physical, or biological agent substances in the air, such as pollutants from vehicles, industrial processes, and natural sources referring to both the indoor and outdoor environment contamination able to modify the natural characteristics of the atmosphere (WHO, 2021). All these components are part of the general external exposome, the set of all measurable exposures at the population level including air pollution or meteorological factors. Outdoor air pollution consists of particulate matter (PM), ground-level ozone (O_3_), sulfur dioxide (SO_2_), nitrogen dioxide (NO_2_), carbon monoxide (CO), and volatile organic compounds (VOCs). Indoor or household air pollution arises from sources like cooking stoves, heating appliances, tobacco smoke, and certain building materials (Tran et al., 2020).

**FIGURE 1:**
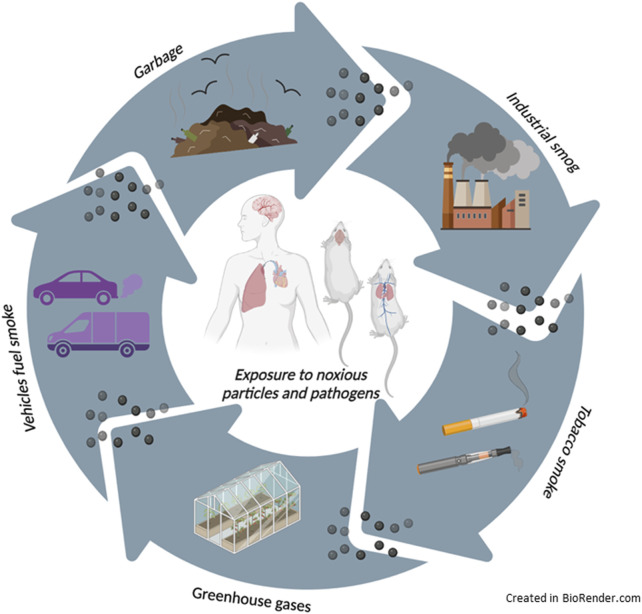
The main sources of particle matter.

Pollution can pass into the atmosphere in many ways, naturally, such as from wildfires or volcanic ash, or anthropogenically, by the emissions from factories, cars, planes, aerosol cans, and cigarette smoke (Figure 1). Prolonged exposure to this kind of pollutant can lead to various health issues, including brain, respiratory, cardiovascular, and brain diseases (Giammona et al., 2024; Manisalidis et al., 2020). Nowadays, outdoor and indoor air pollution represent a global health crisis, causing over 4.2 million deaths annually, with rising morbidity and mortality (Kelly and Fussell, 2015). The WHO declared that most of the world’s habitats live where air quality exceeds limits concerning the annual (Faridi et al., 2023). However, despite many studies on air pollutant-associated adverse health effects, the underlying molecular mechanisms by which air pollutants initiate disease remain mainly unclear (Burkholder et al., 2017).

The most dangerous component among atmospheric pollutants is particulate matter (PM), which refers to tiny particles or droplets in the air that can be inhaled into the lungs. It is classified based on aerodynamic diameters, namely PM2.5 (including particles with a diameter of 2.5 μm or smaller), PM10 (including particles with a diameter of 10 μm or smaller), and submicrometric PM0.1 (particles with a diameter of less than 0.1 µm) (Burkholder et al., 2017; Lara et al., 2023; Zhou et al., 2021).

PMs were recently included within the carcinogen categories, (Harm to human health from air pollution in Europe: burden of disease (Briefing, 2023; Thangavel et al., 2022; Air quality in Europe Report, 2022; Lavigne et al., 2019). Consequently, the monitoring and controlling of PM levels are crucial for air quality management (Air Quality in Europe Report, 2022; Yang et al., 2018) impacting human and planetary health (Cena et al., 2024).

There is much evidence discussing the crucial role of microRNA (miRNA) families in driving the biological responses to pollutant exposure. miRNAs are endogenous non-coding single-stranded small RNA family regulating huge gene pathways and the complex regulatory network generated by miRNA interaction with their target mRNAs plays a key role in regulating several cellular processes. Considering that air pollutant exposure has the potential to change the miRNA profiles, miRNA expression deregulation may be considered as a marker of air pollution effect on human health (Espín-Pérez et al., 2018; Giammona et al., 2024; Krauskopf et al., 2017).

To understand the influence of PM exposure on diseases, also mediated by altered miRNA expression, we have reviewed the main papers discussing the connection between the effect of air pollution and the miRNA involvement, highlighting the importance of animal models to validate this connection. In particular, the use of animals is indispensable to study the effect of air pollution in a complex system to evaluate its *in vivo* distribution through the biological barriers, particularly when we consider the effects on the lungs, brain, and cardiovascular system (Ferreira et al., 2022).

**FIGURE 2:**
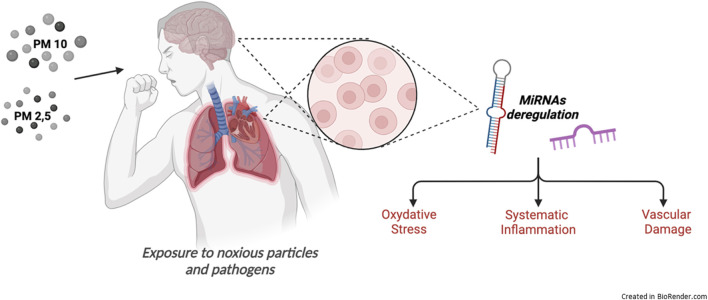
Biological processes induced by air pollution exposure.

The aim of this review focuses on the main biological consequences of PM exposure reporting reliable research developed *in vivo* models and imaging strategies to evaluate PM-related disease, with particular interest in noninvasive Positron Emission Tomography (PET) technique and *ex vivo*/*in vitro* studies, and the related critical gene pathways involved in the control of the lung-brain axis responsive to air pollution, to identify the non-coding RNA families, and miRNAs in particular, responsible for their modulation (Figure 2).

## 2 Methods

**FIGURE 3:**
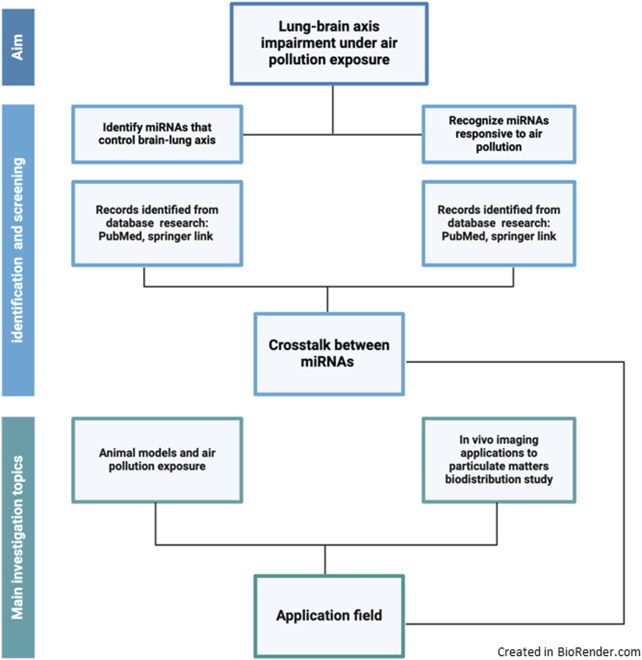
Schematic view describing the selection of the literature strategy used for the review.

To find out a pool of microRNAs involved in the lung-brain axis responding to air quality pollution and/or cause lung cancer, we based on a PubMed search by the keywords “microRNA and lung-brain axis.” The research updated on 23 September 2024 gave 37 papers on PubMed; reviews (Barangi et al., 2023; Casciaro et al., 2020) were excluded, as well as those papers regarding the methylation process (Huang et al., 2023). Then we asked the PubMed database to search for “microRNA and brain and air particulate” (23 September 2024). We obtained 13 papers. From those, reviews and articles not in English (Sagai, 2019) and English regarding nano-drug delivery in Alzheimer’s disease were excluded (Fu et al., 2022). Comparing the two groups of miRNAs along these two metanalytic approaches, we selected common miRNAs that are affected by air pollution exposure and that could be involved in the control of the lung-brain axis. Moreover, by deeply discussing those that exploit mice models, we could focus on PM exposure studies that could be investigated by *in vivo* imaging tools to best highlight the adverse health effects (Figure 3).

## 3 Animal models to study air pollution effects

Animal testing has long been a cornerstone of toxicological research, especially in the evaluation of air pollutant toxicity. Rats and mice, due to their ease of handling and genetic similarity to humans, are frequently chosen as models for such studies (Zavala et al., 2020).

One significant advantage of animal testing is the control of the whole experimental setup (mortality rates, gross abnormalities, organ and tissue-level effects, and molecular-level changes) (Costa et al., 2014). Additionally, animal models can be tailored to specific research questions, such as investigating disease mechanisms or susceptibility to certain conditions or examining critical developmental stages, (i.e., *in-utero* exposure) (Yokota et al., 2013). According to this study, maternal diesel exhaust particle (DEP) exposure may lead to cognitive deficits, particularly in spatial memory, emphasizing the need for further research to develop preventive measures to mitigate the long-term effects of DEP exposure on brain function. Other studies underlie that prenatal exposure to UFPs may also affect fetal health causing asthma in children (Wright et al., 2021).

Despite several advantages to using animal models, there are notable limitations. Physiological and genetic differences between animals and humans can affect how they respond to air pollutants, potentially impacting the relevance of study findings to human health. Moreover, conducting animal studies requires substantial time, resources, and ethical considerations, which have prompted efforts to reduce reliance on animal testing in recent years.

The choice between *in vivo*, *in vitro* and *ex vivo* models in air pollution research is crucial for accurately assessing human health risks. While animal studies provide comprehensive insights into systemic effects, physiological and genetic differences between species (Rydell-Törmänen and Jhonson, 2019) may limit their direct applicability to humans. Additionally, ethical and logistical challenges have led to increased reliance on alternative models. *In vitro* systems (Rothen-Rutishauser et al., 2008), including single-cell cultures, co-cultures, and advanced 3D or organ-on-chip models, offer controlled environments to study cellular responses to pollutants. *Ex vivo* models (Lakhdar et al., 2022), derived from intact tissues, bridge the gap between *in vitro* and *in vivo* approaches by preserving tissue architecture and physiological interactions. Each model has its advantages and limitations, but when used together, they enhance our understanding of pollution-induced toxicity and disease mechanisms.

Nevertheless, animal models remain unique analogs of human behavioral phenotypes that are risk markers for internalizing and externalizing problems (behavioral inhibition, behavioral exuberance, irritability), and to identify commonalities among the neural mechanisms underlying these behavioral phenotypes and the neural targets of air pollutants (polycyclic aromatic hydrocarbons, traffic-related air pollutants, fine particulate matter <2.5 µm) (Farina et al., 2013; Margolis et al., 2022). Some common exposure approaches including intranasal or intratracheal instillation, nose-only inhalation, whole-body exposure, and intravenous injection have been reviewed with a summary of their performance, merit, limitation, and application. Each of these approaches has its own merits, limitations, targeted organs and systems, and requirements for study design (Shang and Sun, 2018).

### 3.1 Exposure methods

There are several routes to expose animals to gaseous or particulate matter-based pollutants; we have chosen to focus solely on those that are physiologically relevant, particularly pulmonary and oral exposures, excluding others such as injection-based methods. These methods, indeed, replicate more realistically the exposure scenarios that reflect how humans and animals naturally encounter airborne pollutants. These routes include oral gavage, aspiration, instillation, and inhalation (Zavala et al., 2020). Gavage is an oral administration in which substances are conveyed to the animals by inserting a small plastic feeding tube through the nose or mouth and into the stomach. The gastrointestinal route of exposure to particulate matter is relevant to explore the toxicity of particles ingested through contaminated food (Danielsen et al., 2008) and water.

Particles can be administered to the airway through nasal or intra-tracheal instillation, and inhalation as airborne aerosol. Pulmonary exposure is particularly relevant, as it directly mimics the inhalation of particulate matter into the lungs, the primary site of deposition for airborne particles. Instillation is a widely used experimental method in toxicity studies, particularly for investigating airborne PM. While the procedure is relatively simple, it typically requires sedation to prevent coughing. The particles are suspended in sterile saline or phosphate-buffered saline (PBS) at the desired concentration before delivery. In nasal instillation, the animal is lightly anesthetized and positioned supine, with the particle suspension delivered dropwise into the nasal cavity using a micropipette. For intratracheal instillation, additional instruments and anesthesia are needed, with particles suspended in saline or PBS and delivered via syringe, needle, endotracheal tube, or catheter (Shang and Sun, 2018). Instillation offers precise dosage but may result in uneven particle deposition and require delivery vehicles (i.e., PBS or other saline solutions) and anesthetization of animals. Inhalation exposure mimics real-life exposure by introducing animals to aerosolized PM. It is more physiologically relevant as it involves natural breathing patterns. However, the setup is more complex and requires controlled conditions (e.g., exposure chambers). Inhalation exposure provides a more natural exposure route (air is the vehicle of delivery) making this method closer to real-life exposure, with direct effects on the respiratory system. However, it requires specialized equipment and special setups to prevent contamination: moreover, it is difficult to control the amount and size of particles reaching the lungs.

It is important to mention that, despite these differences, some studies suggest that exposure to poorly soluble particles via instillation or inhalation yields similar levels for lung toxicity (Møller et al., 2008). Inhalation exposure can be performed through whole-body or nose-only chambers. Nose-only exposure offers advantages such as higher exposure concentration, reduced waste due to a smaller chamber volume, and the ability to assess particle effects solely through the nose route. However, it is necessary to keep animals immobile and restrained in the chamber through rigid or soft nets or tubes during nose-only exposure and this procedure may induce mild stress. In contrast, whole-body exposure minimizes stress other than exposure-related stress, making it suitable for prolonged and repeated inhalation studies. Nevertheless, concerns persist regarding the potential contribution of other exposure routes affecting particle deposition and health outcomes (Oyabu et al., 2016).

The biodistribution of ultrafine particles (UFPs) varies significantly depending on the route of administration. Inhalation is the primary exposure pathway, leading to particle deposition in different regions of the respiratory tract based on size, with PM10 accumulating in the upper airways and smaller particles, such as PM2.5 and UFPs, reaching the alveoli. Once in the lungs, UFPs can translocate into the bloodstream, potentially affecting distant organs like the liver, heart, and brain. In contrast, particles administered via intratracheal instillation may exhibit a different distribution pattern due to direct lung delivery, potentially bypassing initial upper airway deposition. Additionally, nasal exposure can facilitate direct translocation to the brain via the olfactory nerve, whereas ingestion primarily directs particles through the gastrointestinal system before systemic absorption. These differences in biodistribution highlight the importance of considering the exposure route when assessing the health risks associated with particulate matter (Han et al., 2023). The summary of all the methods described is in Table 1.

**TABLE 1:** Summary of administration methods and particle concentration.

Way of administration	Concentration	References
Oral gavage	0.64 mg/kg body weight for 24h	Danielsen et al. (2008)
Nasal or intra-tracheal instillation, and inhalation	4.01 ± 1.11 mg/m^3^ 3. dynamic inhalation chamber with aerosol 20 mg/kg/day	1. Møller et al. (2008) 2. Oyabu et al. (2016) 3. Han et al. (2023)
Intratracheal instillation	Not reported	Shang and Sun (2018)

### 3.2 Time exposure

Animal studies typically employ different time exposures and frequencies, ranging from acute to chronic designs, to assess various toxicological outcomes. The duration and frequency of exposure significantly influence the observed effects, as different exposure designs—acute, subchronic, and chronic—provide distinct insights into toxicity mechanisms. Acute exposures might last for a single day or span up to 2 weeks, sub-chronic might last for 1–3 months and chronic designs extend over longer periods, up to 2 years. Acute exposures primarily reveal immediate physiological or biochemical responses. In contrast, subchronic exposure allows for a better understanding of cumulative toxicity and adaptive biological responses. Chronic studies are essential for assessing long-term health risks. Notably, toxicity resulting from a single high-dose exposure may differ substantially from low-dose repeated exposure, as chronic exposure can lead to bioaccumulation, prolonged inflammation, or delayed onset of adverse effects. Single-dose administration typically yields acute effects, while chronic exposure is associated with long-term effects (Mohammadpour et al., 2019). Thus, selecting the appropriate exposure duration is critical for accurately assessing potential health risks associated with environmental particles.

### 3.3 Translational relevance to human health

Rodent models are valuable tools for studying the effects of air pollutants (for example, related to infectious and allergic lung diseases), helping to establish cause-effect relationships and exposure thresholds. However, extrapolating these findings to human health is complex due to interspecies differences in immune responses and pollutant sensitivity. Improving risk assessment requires mechanistic insights and comparative models to bridge the gap between animal data and human health outcomes (Selgrade, 2000).

## 4 Noninvasive monitoring of air pollution effects

In this section, we comprehensively explore the current imaging strategies employed for monitoring *ex vivo/in vivo* pollutants distribution and relative induced changes in the lung-brain axis, highlighting their applications, limitations, and future directions, with particular focus on Positron Emission Tomography (PET) and Single Photon Emission Computed Tomography (SPECT) imaging.

PET and SPECT molecular imaging are tomographic techniques that, using specific radiopharmaceuticals, can monitor physiological functions, metabolisms and the expression of different markers as neurotransmitters and receptors PET and SPECT have be used to monitor the effect of pollutants on cardiac and respiratory systems and on brain (Zaheer et al., 2018), and could provide quantitative data on the exposure to labeled nanomaterials, including PM, in living subjects. As an example (Nemmar et al., 2002), ultrafine carbon particles (5–10 nm) have been labeled with Tc-99m and administered by aerosol in healthy volunteers (3–5 breaths corresponding to 100 MBq). Images have been acquired using a planar gamma camera, which can register single photon emission deriving from labeled material within the body. In parallel, blood samples were collected via a venous catheter at different times and radioactivity was measured in a gamma counter. Radioactivity analysis evidenced the accumulation of particulate firstly in the lungs and thereafter in the other organs with a mechanism mediated by blood translocation, validating the SPECT as a useful tool to monitor particulate fate. Technological advances led to the development of SPECT/CT hybrid tomographs with improvement in spatial resolution and sensitivity (Ljungberg and Pretorius, 2018). As for PET and PET/CT systems, the amount of radioactivity can be expressed as counts per minute (CPM) or converted to standardized uptake values (SUV), considering body mass and administered radioactivity. Tissue radioactivity can be measured and compared across different regions (Vaquero and Kinahan, 2015).

### 4.1 Particulate matter biodistribution study by *in vivo* imaging

**FIGURE 4:**
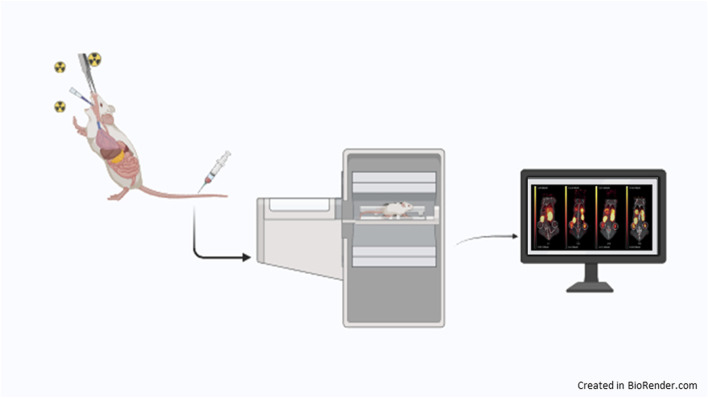
Radioisotope labeled PM can be administered in mice via intratracheal, oral, or intravenous injection to monitor the biodistribution of PM using preclinical PET/CT system.

A significant challenge in comparing health risks associated with micro or nano-toxic particles lies in accurately measuring these hazardous materials within biological tissues. Various methods, including the use of fluorescent tags and mass analysis, have been explored to investigate the biological uptake of PM in living subjects (Jiang et al., 2021; Li et al., 2019). While these methods offer valuable insights into the distribution and enable tissue analysis of carbonaceous particles, they reported some important limitations. Optical imaging and fluorescent labeling techniques, for instance, often encounter difficulties in accurately measuring target analytes in deep tissues due to limited signal penetration (Li et al., 2019; Son et al., 2022). Additionally, mass analysis necessitates intricate pretreatment steps, and it is ill-suited for quantitatively measuring PM across a wide size range within biological tissue (Yuan et al., 2020). An alternative approach for *in vivo* tracking of carbonaceous matter involves the development of a radiotracer by radioisotope-based labeling of PM (Figure 4). This method facilitates precise determination of uptake and tissue distribution without the need for pretreatment of tissue sections, as required *in vitro/ex vivo* (Bartels et al., 2020). Furthermore, it enables noninvasive imaging of PM in organs of interest using nuclear imaging tools like PET or SPECT, allowing for direct quantification of the molecule’s concentration in living biological systems (Im et al., 2022; Lee et al., 2020). Therefore, the use of radiotracers helps overcome the limitations of conventional analytical methods. Previous studies have demonstrated the preparation of radioisotope-labeled particulate matter with a sub-micrometer size distribution using radioactive iodine (^125^I) (Lee et al., 2019), enabling visualization of harmful carbonaceous matter *via* in tracheal instillation in mice. Authors assessed that ^125^I particles retained their stability *in vivo*, which allowed a reliable determination of the ^125^I particles biodistribution, with little release of ^125^I from particles (Cavina et al., 2017). In a recent study (Park et al., 2023), the authors employed ^89^Zr-tagged pyrene (Dilworth and Pascu, 2018; Lee et al., 2019) as a radiotracer to prepare ^89^Zr radioisotope-incorporated PM, suitable for PET imaging, which provides spatiotemporal information over several days (van Dongen et al., 2021). The authors tested three different routes of administration: intratracheal, oral, and intravenous injection and results reported that PM was largely distributed in the lungs and only slowly cleared after 7 days in mice exposed via the intratracheal route. In addition, the uptake of ^89^Zr-PM was visible also in other organs, such as the heart, spleen, and liver. Uptake values in these organs were also noticeable following exposure via the intravenous route. In contrast, most of the orally administered PM was excreted quickly within a day. These results suggest that continuous inhalation exposure to PM causes serious lung damage and might cause toxic effects in the extrapulmonary organs such as the brain (Yuan et al., 2024). Furthermore, the authors developed a method that can be used in future studies focused on the analysis of the *in vivo* behavior of hazardous carbonaceous matter. In fact, ^89^Zirconium possesses unique physical and chemical properties such as a long half-life that provides information on long-circulating molecules and their pharmacokinetics, and a favorable emission energy that is lower than some other PET isotopes (e.g., ^18^F) contributing to high-resolution imaging and improved quantitative accuracy in PET scans. Moreover, ^89^Zr can be efficiently conjugated to biomolecules by deferoxamine (DFO) as a chelator that produces a stable complex, with low off-target radiation and increases imaging specificity. Thus, PET/SPECT imaging could represent a powerful tool for non-invasively monitoring the *in vivo* distribution of PM with high-resolution and quantitative data (Pan et al., 2023; Zaheer et al., 2018).

### 4.2 *In vivo* PET/SPECT molecular imaging applications for monitoring air pollution-induced neuropathological alterations

Growing evidence suggests that exposure to air pollution is linked to various central nervous system (CNS) disorders via the lung-brain axis (Block et al., 2012; Block and Calderón-Garcidueñas, 2009). Living in areas with heightened urban air pollution levels has been linked to reduced cognitive function in older individuals, (Power et al., 2013, 2011; Wellenius et al., 2012), increased risk of autism (Becerra et al., 2013; Roberts et al., 2013; Volk et al., 2013, 2011), Alzheimer’s disease (AD) (Jung et al., 2015) and Parkinson’s disease (PD) (Kirrane et al., 2015) hastened disease progression leading to initial hospitalization in neurodegenerative disorders (Kioumourtzoglou et al., 2016), such as AD-like (Calderón-Garcidueñas et al., 2012; Calderón-Garcidueñas et al., 2008; Calderón-Garcidueñas et al., 2004) and PD-like (Calderón-Garcidueñas et al., 2013; Calderón-Garcidueñas et al., 2011) neuropathology in humans, as well as greater incidence of stroke (Bedada et al., 2012; Wellenius et al., 2012). While the specific mechanisms remain unclear, there is growing support for the theory that neuroinflammation and microglial activation with the consequent hampering of neurovascular unit integrity, serve as a common mechanism through which air pollution impacts these various CNS conditions. Amyloid-β (Aβ) deposition is a main feature of Alzheimer’s disease (AD) and may be promoted by exogenous factors, such as ambient air quality. Both PM2.5 and O3 play significant roles in the global burden of disease and mortality (Cohen et al., 2017; Landrigan et al., 2018), as evidenced by various studies. They have been linked to an elevated risk of cognitive decline, clinically diagnosed Alzheimer’s disease (AD), and all-cause dementia in epidemiological investigations (Block et al., 2012; Costa et al., 2020; Jayaraj et al., 2017; Paul et al., 2019; Peters et al., 2019; Russ et al., 2019; Tsai et al., 2019). Recent updates from the Lancet Commission in 2020 on dementia prevention, intervention, and care have highlighted exposure to air pollution as a modifiable risk factor for cognitive decline in later life (Bhatt et al., 2015; Cacciottolo et al., 2020; Cacciottolo et al., 2017; Durga et al., 2015; Hullmann et al., 2017; Jang et al., 2018; Levesque et al., 2011a; Livingston et al., 2020). Human studies assessing neuropathological or cerebrospinal fluid levels of Aβ1-42 have observed that individuals residing in more polluted areas, including children, young adults, and middle-aged adults, are more likely to exhibit signs of altered Aβ processing and, in some cases, pathological amyloid deposition (Calderón-Garcidueñas et al., 2020; Calderón-Garcidueñas et al., 2018; Calderón-Garcidueñas et al., 2016a; Calderón-Garcidueñas et al., 2012; Calderón-Garcidueñas et al., 2008; Calderón-Garcidueñas et al., 2004). Recently, in a cross-sectional study of 178 individuals with cognitive impairment (Iaccarino et al., 2021), it was observed that people residing in regions with inferior air quality displayed a heightened likelihood of exhibiting positive amyloid positron emission tomography scan outcomes using 1 of 3 most common Aβ fluorine-18 labeled tracers (^18^F-florbetapir, ^18^F-florbetaben, or ^18^F-flutemetamol) (Iaccarino et al., 2021). Specifically, high levels of PM2.5 concentrations were linked to the presence of amyloid-β plaques in the brain, which are indicative of AD. This correlation exhibited a proportional relationship with the dosage of exposure and remained statistically significant following adjustments for demographic, lifestyle, socioeconomic factors, and medical comorbidities. The conclusions drawn from this research indicate that exposure to air pollution is correlated with the development of amyloid-β pathology in older adults grappling with cognitive impairment (Iaccarino et al., 2021). In addition, these results confirmed that PET imaging serves as a fundamental technique for *in vivo* assessment of Aβ brain accumulation (amyloid PET scan) even air pollution correlated.

Similarly, a population-centric investigation carried out in Canada reported that exposure to air pollutants, particularly PM2.5, is associated with the onset of PD (Shin et al., 2018). Further investigations also confirmed that prolonged exposure to air pollution elevates the probability of PD (Hu et al., 2019). Accordingly, a recent longitudinal cohort exploration by Liuhua Shi et al. revealed that for every 5 mg/m^3^ rise in yearly PM2.5 concentration, the hazard ratio stood at 1.13 for the initial hospitalization for PD, suggesting a significant correlation between exposure to annual mean PM2.5 and heightened PD risk (Shi et al., 2020). Studies from both animal experiments and humans have supported the hypothesis that pathological α-syn can accumulate in the gut, spread to the brainstem by the vagus nerve, and eventually induce neuronal loss in the nervous system SN (Shannon et al., 2012). Little direct evidence sustains air pollutants can induce α-syn aggregates in the gut, which spread to the central nervous system (CNS). There is an increasing body of publications demonstrating that air pollutants can change the gut mucosa, which is thought to promote α-syn pathology (Murata et al., 2022). In animal studies, α-syn preformed fibrils injected into the duodenum induce α-syn spread into brainstem nuclei and then to the SN (Kim et al., 2019). Although PET imaging approach has not been applied yet to investigate the α-syn accumulation in correlative studies of air pollution and PD onset, the DaTscan PET imaging technique is sensitive enough to detect presynaptic dopamine neuronal dysfunction and could be considered as one of useful *in vivo* diagnostic tools for the early detection of degenerative Parkinsonism (Bega et al., 2021). Air pollution exposure has been evidenced to induce inflammation and leakiness in the gut which may be a trigger for α-syn aggregates and alter the risk of inflammatory bowel disease (IBD) (Murata et al., 2022). Indeed, most studies focusing on the mechanisms of air pollution-induced adverse effects have predominantly investigated its role in causing inflammation (Jayaraj et al., 2017). CNS inflammation and oxidative stress are significant findings in the brains of individuals with PD and AD, and air pollution seems to exacerbate these conditions. Evidence from human autopsy studies and rodent experiments supports that air pollution heightens inflammation within the CNS (Calderón-Garcidueñas et al., 2008; Calderón-Garcidueñas et al., 2004; Jayaraj et al., 2017). Air pollution consists of a diverse blend of gases, PM, and smaller chemical entities. Some of these substances can enter the brain either through the bloodstream or by directly diffusing through the olfactory system. In this pathological process the Neurovascular Unit (NVUs) integrity seems to have played a pivotal role. The NVUs consist of endothelial cells with tight junctions, a basement membrane, and perivascular glial sheets. Maintaining the stability of NVUs is crucial for brain health, and disruptions in these units are associated with neurodegenerative diseases such as AD and PD (Calderón-Garcidueñas et al., 2016b). Children who are exposed to air pollution over their lifetime show notable increases in serum of high-affinity antibodies targeting tight junctions and neural proteins and possess cerebrospinal fluid (CSF) antibodies against myelin basic protein (Calderón-Garcidueñas et al., 2014). Additionally, the expression of cyclooxygenase-2 (COX2) and interleukin-1B (IL-1B) is elevated in both the olfactory bulb and frontal cortex (Calderón-Garcidueñas et al., 2007) with an accumulation of beta-amyloid peptide (Aβ42) within the frontal cortex (Calderón-Garcidueñas et al., 2007). Likewise, several studies have indicated that central nervous system inflammation occurs in rodent models, mirroring the inflammation observed in human PD brains. Notably, it has been found that diesel exhaust (DE) exposure elevates the expression of certain inflammatory genes in the olfactory bulb (OB) of mice, an area of the brain where PD pathology manifests early signs of disease (Levesque et al., 2011a, 2011b; Yokota et al., 2013). Similarly, inflammatory changes have been detected in the brains of dogs and humans living in urban environments compared to those in rural areas, with researchers attributing these changes to higher levels of air pollution (Calderón-Garcidueñas et al., 2008; Calderón-Garcidueñas et al., 2004; Calderón-Garcidueñas et al., 2003). Although these studies have certain limitations, they collectively suggest a causal relationship between air pollution and an increased risk of neurodegeneration through mechanisms of direct neurotoxicity and/or neuroinflammation. Therefore, for *in vivo* neuroinflammation assessment, an important contribution should be offered by the different advanced PET radiotracers targeting the potential molecules in the neuroinflammation process (Choi et al., 2009). Cyclooxygenase (COX) is crucial in the production of prostaglandin H2, which serves as a precursor for prostaglandins and thromboxane. There are two COX isoforms, COX-1 and COX-2, which play significant roles in neuroinflammation and are associated with various neurodegenerative diseases, particularly AD. Immunochemical evidence has shown that both COX-1 and COX-2 are present in microglia and neurons within the CNS (Choi et al., 2009). Various radiotracers for COX-1 and COX-2 have been well established, such as ^18^FTMI, ^18^F-triacoxib, ^11^C-rofecoxib, ^11^C-KTP-Me, ^11^C-PS13, and ^11^C-MC1. Among these, the ^11^C-KTP-Me showed the most promising results (Choi et al., 2009; Horti et al., 2019; Kumar et al., 2018; Ohnishi et al., 2014; Zhou et al., 2021). Several reports indicate that also the 18 kDa Translocator Protein (TSPO), involved in a range of cellular activities such as cholesterol transport and hormone synthesis, plays a fundamental role in inflammatory response. However, its precise role in brain inflammation remains unclear. Under normal conditions, TSPO expression is low in CNS microglia, but it significantly increases following neuroinflammation, correlating with the abnormal activation of microglia (Li et al., 2016). Immunohistochemistry staining has shown that TSPO upregulation and microglia activation spatially coincide post-neurotoxic intervention, implying that TSPO can serve as a marker for activated microglia and a potential method to assess neuroinflammation (Cosenza-Nashat et al., 2009). Thus, imaging agents targeting TSPO represent *in vivo* biomarkers for microglia activation and neuroinflammation. Furthermore, in a preclinical study by Toby B. Cole et al., acute exposure of C57BL/6 mice to diesel exhaust (DE) caused significant increases in lipid peroxidation and pro-inflammatory cytokines (TNF-α, IL-1β, IL-3, IL-6, IL-1α) in various brain areas (particularly olfactory bulb and hippocampus) (Cole et al., 2016). DE exposure also caused activation of microglia, as measured by increased Iba1 (ionized calcium-binding adaptor molecule 1) and TSPO (translocator protein) expression, which correlated with increased uptake of [^3^H]-DPA_713 as a radiolabeled ligand to quantify TSPO levels *ex vivo* by autoradiography. The ^11^C-PK11195 radioligand, a high-affinity TSPO ligand, was the first probe developed for PET imaging of neuroinflammation (Cagnin et al., 2001). The inherent properties of the compound and the complexity of carbon-11 radiolabeling within a brief 20-minute timeframe hindered the advancement of this technique for investigating neuroinflammation. Additionally, due to inadequate blood-brain barrier (BBB) penetration and minimal brain uptake, ^11^C-PK11195 exhibits a low signal-to-noise ratio (Ching et al., 2012; Lockhart et al., 2003). These shortcomings have led to the development of advanced-generation of TSPO radiotracers, such as ^18^FGE-180 (R, S)-^18^F-GE-387, ^11^C-PBR28, ^18^F-DPA-714, ^18^F-VC701 to improve the bioavailability, an enhanced signal-to-noise ratio with higher binding affinity compared to ^11^C-PK11195 (Belloli et al., 2018; Di Grigoli et al., 2015; Hamelin et al., 2018, 2016; Kreisl et al., 2010).

Overall, PET/SPECT imaging provides real-time *in vivo* distribution data of radiolabeled-PM and simultaneously represents an investigation tool for the PM-induced alterations in the lung-brain axis. By elucidating the mechanisms underlying these effects, PET imaging could contribute to the understanding of air pollution-related health risks and facilitate the development of targeted interventions. Nonetheless, PET/SPECT application requires specific radiolabeled PM tracers which are expensive, and require specialized equipment, trained personnel, and access to radiopharmaceuticals (Zaheer et al., 2018). The selection of a tracer significantly affects detection sensitivity and specificity, meaning that not all types of PM can be efficiently labeled and monitored. Additionally, some radioisotopes, such as ^18^F and ^64^Cu, have a short half-life, requiring immediate use post-synthesis, which complicates large-scale or longitudinal studies. While PET imaging offers excellent sensitivity, its spatial resolution (around 1 mm) is lower compared to techniques like MRI or CT. Furthermore, SPECT generally has an even lower resolution than PET, making it more challenging to accurately assess PM accumulation in small anatomical regions. Although dynamic imaging is feasible, the time resolution is influenced by the tracer’s decay and the imaging protocol, potentially limiting real-time tracking (Meikle et al., 2006).

## 5 PM-respondent non-coding RNA along the lung-brain axis

**FIGURE 5:**
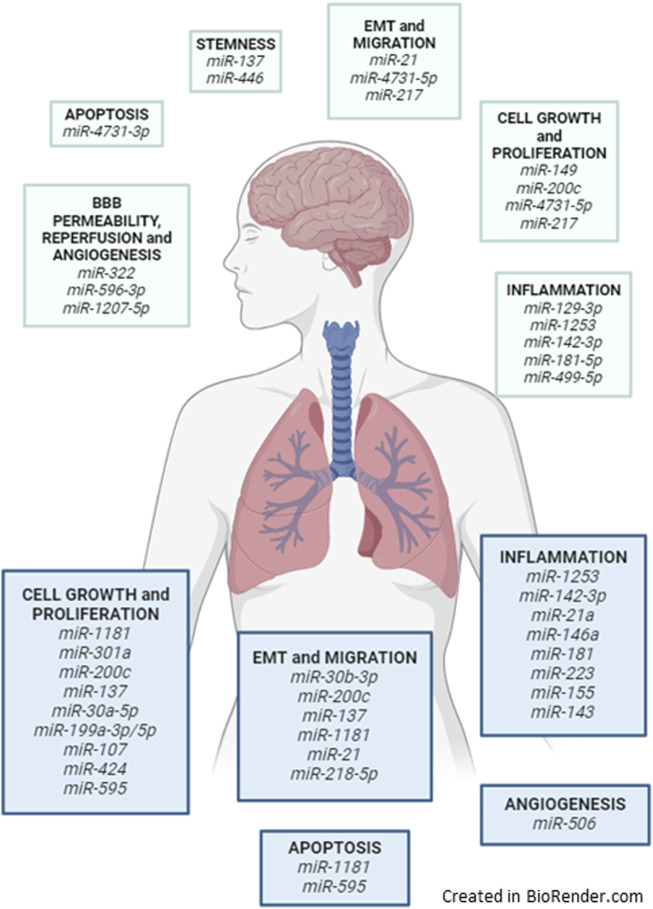
Lung-brain axis and the miRNAs discussed and related biological processes when exposed to airborne pollutants.

This review by literature query explores key miRNAs in lung cancer and brain metastases, highlighting their role in tumor progression, metastasis, metabolism, immune modulation, and vascular remodeling (Figures 3, 5).

### 5.1 miRNAs are involved in cell growth and metastasis control

Our research reports as main molecular regulators of the lung-brain axis, the miR-200c and miR-149.

#### 5.1.1 miR200c

It regulates the epithelial-mesenchymal transition (EMT) process and controls brain metastasis formation from primary tumors of the breast, melanoma, and the gastrointestinal tract. In addition, it is responsible for the regulation of EpCam adhesion molecules in glioma cells where highly expressed promoting proliferation and tumorigenesis (Cavallari et al., 2021; Fotakopoulos et al., 2023; Xue et al., 2021). This miRNA also belongs to the miR-200c/AUF1/SOX2/miR-137 axis: under the control of JUN transcription factor and the lncRNA MEG3 regulation, it promotes the transformation of malignant bronchial epithelial cells after nickel exposure (Yang et al., 2024).

#### 5.1.2 miR-149

It controls the expression of lncRNA BCYRN1 and acts as an oncogene in NSCLC regulating the aerobic glycolysis targeting pyruvate kinase M1/2 (PKM2). Inhibition of miR-149 enhanced PKM2, modulating glucose consumption, while the silence of BCYRN1 affected lactate production (Lang et al., 2020).

Regarding brain metastases, other miRNAs could be involved, such as *miR-522-3p,* which can regulate Tensin-1 expression, helping tumoral cell invasion by altering BBB permeability through ZO-1 and OCLN expression (Liu et al., 2024). *MiR-30a-5p* promotes proliferation in lung adenoma by regulating ABL2 expression and activating the PI3K/AKT pathway (Miao and Liu, 2023; Yang et al., 2021). In NSCLC, *miR-199a-3p/5p,* regulates mTOR signaling promoting cell migration and invasion (Liu et al., 2022; Song et al., 2022), and *miR-107* and *miR-595,* control the metastasis and cell proliferation modulating the brain-derived neurotrophic factor (BDNF) expression and E2F7 axis (Bai et al., 2020; Hong et al., 2020).

In the human pulmonary adenocarcinoma brain metastasis cellular model, two miRNAs can be considered: *miR-217* act as metastasis suppressor targeting the expression of sirtuin 1, reducing the proliferation, migration, and invasion (Jiang et al., 2021); *miR-574-5p,* which is under the control of lncRNA LM2-4175 and linc-ZNF469-3, targets ZEB1 expression (Wang et al., 2018).

All these miRNAs are potentially involved in the control of lung cancer cell proliferation and invasion, two processes that could be monitored by PET imaging.

### 5.2 miRNAs involved in stemness control in the lung-brain axis

*MiR-1237* controls both myocyte enhancer factor-2A (MEF2A) and the pluripotency transcription factor OCT4 and SOX2 (Channakkar et al., 2020).

*miR-4466* is found in chronic nicotine exposure activated STAT3-driven N2 neutrophils, where it induces secretion to promote stemness via the SKI/SOX2/CPT1A axis (Tyagi et al., 2022). In these cases, these two miRNAs could be initially responsive to smoking-derived PM, and then, once released in the blood, alter functional pathways in the brain.

### 5.3 miRNAs controlling inflammation in the lung-brain axis

In this group, there are multiple miRNAs modulating inflammatory responses. *miR-21a, miR-146a, miR-181, miR-223, miR-222-3p*, *miR-155* and *miR-143* emerged even as regulators of immune response pathways and are all decreased by positive Lactobacilli and Bifidobacteria exposure (Casciaro et al., 2020; Li et al., 2024). miR-142-3p was described as a TGF-β1 regulator by driving microglial M2 polarization and its suppression leads to brain metastasis of NSCLC (Xu et al., 2023).

Among the other inflammatory miRNAs, *miR-1253* regulates IL-6 expression and cisplatin resistance in NSCLC, while acting as a tumor suppressor in medulloblastoma and neurological disorders (He and Li, 2024). The same miRNA shows tumor suppressor activity in medulloblastoma by regulating ferroptosis, and cisplatin response, but also neurological disorders, such as Alzheimer’s disease (Kanchan et al., 2018; Kavoosi et al., 2024). *mir-301* could be a target of inflammatory pathways activation by multiple inflammatory stimuli, such as TGFb stimulus and IL-6, which controls fibrosis through the mammalian target of rapamycin (mTOR) signaling pathway and consequently might serve as a potential therapeutic target (Wang et al., 2020). *miR-596-3p* is reported to be upregulated in primary metastatic tumors from NSCLC, while RNA-Seq data of brain metastasis cells revealed its downregulation (Li et al., 2022). This miRNA seems to be involved in the regulation of two key genes for brain metastasis formation, YAP1, and IL-8, able also to restrain the permeability of the BBB. This lncRNA acts as a sponge for *miR-1207-5p*, leading to the expression of EPB41L5 mRNA (Wu et al., 2021; Li et al., 2022).

In glioma, stem cells communicate with microglia via miRNA in extracellular vesicles, with *miR-129-3p* regulating IL-6, IL-8, and TNFα. mir-301 could be activated as targets of inflammatory pathways activation by multiple inflammatory stimuli such as TGFβ stimulus and IL-6, which controls fibrosis through the mammalian target of rapamycin (mTOR) signaling pathway and consequently might serve as a potential therapeutic target (Wang et al., 2020). In glioma, stem cells communicate with microglia via miRNA in extracellular vesicles, with miR-129-3p regulating IL-6, IL-8, and TNFα (Yang et al., 2020). miRNAs regulate the inflammatory response to viral infections. In EV71 infection, miR-155-5p is upregulated, influencing EV71 titers, IFN1 production, and mouse survival via the FOXO3/miR-155-5p/IRF7 axis (Yang et al., 2020). miRNAs regulate the inflammatory response to viral infections. In EV71 infection, *miR-155-5p* is upregulated, influencing EV71 titers, IFN1 production, and mouse survival via the FOXO3/miR-155-5p/IRF7 axis (Yang et al., 2020).

### 5.4 MiRNA with a role in brain metastases and angiogenesis

Lung carcinoma expresses *miR-21*, regulating macrophage polarization and EMT via ERK/STAT3, and *miR-218-5p*, targeting TRIM9, influence vascular permeability, synapse organization, and neuron development (Yoshino et al., 2022; Tiong et al., 2023; Barangi et al., 2023; Dai et al., 2017).

*miR-506* downregulation increases the expression of STAT3, leading to VEGFα induction, thus contributing to angiogenesis (Wang et al., 2021). VEGF and vascular permeability are also target of *miR-424/322*, which modulates the hypoxia response in high-altitude pulmonary edema. Hypoxia disrupts endothelial junctions via HIF1a, increasing cerebral edema risk. MiR-424/322 counteracts this, reducing vascular leakage (Tsai et al., 2019).

Regarding vascular remodeling, PDGFBB promotes the proliferation and migration of human pulmonary arterial smooth muscle cells via regulating the expression of *miR-1181*; the PDGF receptor/PKCa was found to silence miR-1181, influencing proliferation and causing vascular remodeling (Qian et al., 2018).

Therefore, the effect of miRNA modulation, due to PM exposition, at the brain level may even not be direct with their target within the tissue; indeed, the release of lung and gut miRNAs into the blood and their modulation by lnc-RNAs and pro-inflammatory molecules leads to a distal modulation of microglia at the CNS level.

All the discussed miRNAs with their described biological functions are described in Table 2 and illustrated in Figure 5.

**TABLE 2:** Lung-brain axis *miRNAs.*

Function	Tissue	miRNA	Axis or target	References
Cell growth and proliferation	Glioma cells	*miR-149*	miR-149/PKM2 and BCYRN1	Lang et al. (2020)
		*miR-200c*	lncRNATCF7/miR-200c/EpCam	Zhao et al. (2018)
		*miR-4731-5p*	FLVCR1/miR-4731-5p/E2F2	Yan et al. (2020)
	Human pulmonary adenocarcinoma brain metastasis	*miR-217*	miR-217/sirtuin 1/p53/KA1	Jiang et al. (2021)
	Lung	*miR-200c, miR-137*	miR-200c/AUF1/SOX2/miR-137	Cavallari et al. (2021), Fotakopoulos et al. (2023), Xue et al. (2021)
		*miR-30a-5p*	miR-30a-5p/ABL/PI3K/AKT	Miao and Liu (2023)
	Lung fibrosis	*miR-301a*	TGFb/IL6/STAT3/miR-301a/TSC1/mTOR	Wang et al. (2020)
	NSCLC	*miR-30a-5p*	circRNA_102481/miR-30a-5p/ROR1	Yang et al. (2021)
		*miR-199a-3p/5p*	Rheb	Xu et al. (2023)
		*miR-107*	circHIPK3/miR-107/BDNF	Hong et al. (2020)
		*miR-595*	RNA circ_0109320/miR-595/E2F7	Bai et al. (2020)
EMT, migration and metastasis	Brain metastasis of NSCLC	*miR-21*	Mir_21/ERK/STAT3	Tiong et al. (2023)
	Human pulmonary adenocarcinoma brain metastasis	*miR-217*	miR-217/sirtuin 1/p53/KA1	Jiang et al. (2021)
	Lung	*miR-200c, miR-137*	miR-200c/AUF1/SOX2/miR-137	Cavallari et al. (2021), Fotakopoulos et al. (2023), Xue et al. (2021)
	NSCLC	*miR-199a-3p/5p*	Rheb	Xu et al. (2023)
		*miR-30b-3p*	ROCK1	Song et al. (2022)
		*miR-218-5p*	miR-218-5p/TRIM9	Barangi et al. (2023), Wang et al. (2018)
	Triple negative breast cancer	*miR-574-5p*	LM2-4175 and linc-ZNF469-3/miR-574-5p/ZEB1	Wang et al. (2018)
	Pulmonary artery smooth muscle cells	*miR-1181*	PDGFR/PKCb/miR-1181/STAT3	Channakkar et al. (2020), Qian et al. (2018)
			PDGFBB/miR-1181/PDGF receptor/PKCb	
Stemness	Neuronal cells	*miR-137*	miR-137/MEF2A/OCT4/SOX2	Channakkar et al. (2020)
	Brain metastasis	*miR-4466*	miR-4466/SKI/SOX2/CPT1A	Tyagi et al. (2022)
Apoptosis	Glioma tissue	*miR-4731-5p*	FLVCR1/miR-4731-5p/E2F2	Yan et al. (2020)
	Pulmonary artery smooth muscle cells	*miR-1181*	PDGFR/PKCb/miR-1181/STAT3	Qian et al. (2018)
	NSCLC	*miR-595*	RNA circ_0109320/miR-595/E2F7	Bai et al. (2020), Yang et al. (2020)
Inflammatory pathway	Glioma	*miR-129-3p*	MALAT1/miR-129-3p/IL-6/IL-8/TNFa	Yang et al. (2020)
	Stroke	*miR-181-5p*	MALAT1/miR-181c-5p	Cao et al. (2020)
	NSCLC brain metastasis	*miR-142-3p*	miR-142-3p/TGFb	Xu et al. (2023)
	NSCLC; medulloblastoma	*miR-1253*	has_circ_0000190/miR-1253/IL-6	He and Li (2024), Kanchan et al. (2018)
	Gut tissue/microbiota	*miR-21a, miR-146a, miR-181, miR-223; miR-155, miR-143*	Th1/TH2 modulators	Casciaro et al. (2020)
	Viral infection	*miR-155-5p*	miR-155-5p/INF1; FOXO3/miR-155-5p/IRF7/INFa-b	Yang et al. (2020)
BBB permeability, reperfusion and angiogenesis	Brain metastasis of lung cancer	*miR-424* *miR-322*	miR-424/-322/HIF1a/VEGF	Tsai et al. (2019)
	NSCLC brain metastasis	*miR-596-3p*	YAP/IL-8	Li et al. (2022)
	NSCLC brain metastasis NSCLC	*miR-1207-5p*	MMP2-2/miR-1207-5p/EPB41L5	Wu et al. (2021)
		*miR-506*	miR-506/STAT3/VEGFa	Wang et al. (2021)

### 5.5 Brain miRNA linked to PM exposure

To find out miRNAs involved in the response of the brain to PM, the PubMed research (23rd September 2024) for the terms “microRNA and brain and particulate matter”; indicates 9 main articles [reviews were excluded (Patsouras and Vlachoyiannopoulos, 2019)].

Cigarette chemicals cause damage to nervous cells leading to the activation of *miR-153-3p*, targeting the PI3K/GSK3b pathway, or *miR-143-3p*, controlling insulin sensitivity (Sun et al., 2023). In a rat model of cerebral ischemia, gallic acid reduced cognitive impairment and neuronal cell death by lowering *miR-124* expression after particulate exposure (Bavarsad et al., 2023). In a rat model of cerebral ischemia, gallic acid reduced cognitive impairment and neuronal cell death by lowering *miR-124* expression after particulate exposure (Bavarsad et al., 2023).

PM2.5 exposure increases Alzheimer’s risk, worsening brain damage in Amyloid-β Transgenic Mouse Models (APP) mice compared to filtered air. It elevates IL-6, TNFα, Aβ-42, and AChE levels while altering multiple miRNAs, such as *miR-193b-5p, 122b-5p, -466h-3p, -10b-5p, -1895, -394-5p,* and *-6412*. Among all, the axis *miR-125b*/Pcdhgb8 and *miR-466h-3p*/IL-17Ra/TGFbR2/A 1-42/AChE were related to PM2.5 exposure (Fu et al., 2022). In bronchial epithelial cells, PM2.5, diesel-exhausted particles PM2.5, and diesel-exhausted particles exposure silenced the *miR-345-5p* by the overexpression of lncRNA SOX2 overlapping transcript (SOX2-OT) and finally targeting the EGFR pathway in lung cancer development (Fu et al., 2021).

In a mouse model of atherosclerosis, the exposure to particulate induced inflammation and nitrate stress, increasing both protein expression (IL-6, MCP-1, p47phox, and 3-NT levels) in serum and circulating miRNAs (*miR-301b-3p*, *let-7c-1-3p*) (Sanchez et al., 2020).

A mouse model that mimics neurovascular conditions using uranium mining dust combined to AirCARE1 mobile inhalation laboratory (Sanchez et al., 2020) found twenty-seven miRNAs linked to altered cellular functions. Nine of these were also present in the serum of the same mice: *let-7a-5p, miR-143-3p, miR-151-5p, miR-28a-3p, miR-322-3p, miR-378c, miR-425-5p, miR-7a-5p, and miR-874-5p*. These miRNAs target key pathways related to signaling, cell guidance and endothelial electrical resistance (Sanchez et al., 2020).

Chronic prenatal exposure to PM2.5 high dosage increased apoptosis in neurons and astrocytes of the hippocampal and cortex regions (Yao et al., 2014). Lifelong exposure to PM0.2 reduced the number of newborn neurons in adult male rats, showing contextual memory defects and depressive behavior. Neuroinflammation was observed mainly in males upon prenatal and neonatal PM exposure, with microglia activation and astrogliosis; these effects are possibly linked to the *miR-9* release, also in the microenvironment and the extracellular vesicles, as well as by the expression of *miR-128, miR-302, let-7* and *miR-9*, involved in the regulation of neural precursor proliferation and neurogenesis (Yao et al., 2014), or *miR-21*, *miR-9, miR-200, miR-17, miR-7, miR-302c,* limiting differentiation process of oligodendrocytes.

**FIGURE 6:**
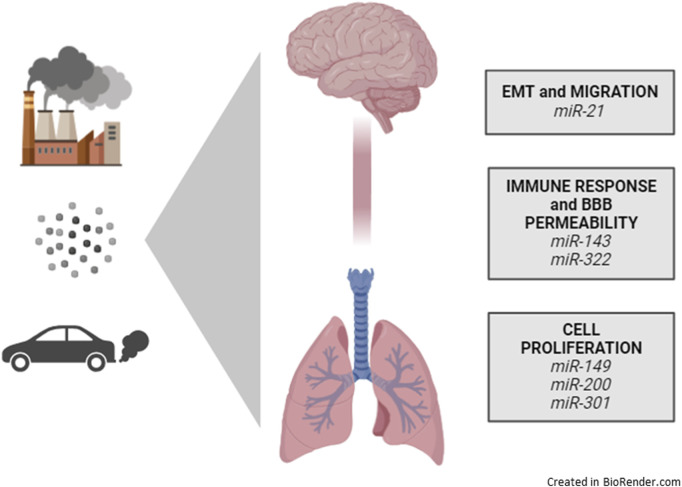
The miRNAs induced by particulate exposure in the lung-brain axis model.

Studies on rat embryos exposed to particulate PM2.5 matter *in utero* (Li et al., 2019) revealed three main circRNAs (circ_015003, circRNA_030724, circ_127215) that participate in the development of the congenital defect in the segmentation process. In particular, the indicated circRNAs sponge the expression of a main group of miRNAs (*miR-214-3p, miR-6334, miR-1839, miR-149-5p, miR-667, miR-3548, miR-139-5p, miR-6324, miR-541-5p, miR-26b, miR-449c, miR-6332, miR-134-3p, miR-3065-3p, miR-105, miR-133c, miR-448-5p, miR-3072, miR-7a-2-3p*) (Li et al., 2019). All the described miRNAs revealed that the brain, as well as the lung, could respond to PM exposure by modulating several classes of lncRNA and miRNAs in particulate, healthy, and pathological subjects, altering specific pathways summarized in Table 3 and Figure 6.

**TABLE 3:** Lung-Brain miRNAs regulated by particulate exposure.

Function	Type of exposure	Tissue	miRNA	Axis or target	References
Insulin resistance and neurotoxicity	0–100–200–300 ng/m^3^ from cigarette smoke Chronic exposure via whole body system	Brain tissue in C57BL/6 mice model	*miR-153-3p*	PI3K/GSK3b and p-Tau	Sun et al. (2023)
Neuronal inflammation	2000–8000 g/m^3^ from dust storm 60’/day for 10 days in a dust storm chamber	Brain tissue in Wistar rats	*miR-124*	Gallic acid/miR-124	Bavarsad et al. (2023)
	61 μg/m^3^ of PM_2.5_ 8 weeks via real world exposure system	Wild type and APP/PS1 transgenic mice	*miR-125b, 466h-3p, 193b-5p, 122b-5p, -10b-5p, -1895, -394-5p, and -6412*	miR-125b/pcdhgb8 and miR-466h-3p/IL-17Ra/TGFbR2/Ab-42/AChE	Fu et al. (2022)
	93,22 μg/m^3^ of PM_2.5_ Acute exposure for 6 days via inhalable intratracheal instillation	Atherosclerosis model in Kunming mice and Apoe deficient mice	*miR-301b-3p, let-7c-1-3p*	miR-301b-3p, let-7c-1-3p/Smad2/3 and TGFβ	Li et al. (2021)
Migration capability of the cells	500 μg/mL PM_2.5_ mixture, 50 μg/mL Diesel exhaust particles, and 100 μg/mL Al2O3 NPs for 24	Human bronchial epithelial (HBE) cells	*miR-345-5p*	SOX2-OT lncRNA/miR-345-5p/EGFR pathway	Fu et al. (2021)
BBB permeability	96.6 ± 60.4 μg/m^3^ whole-body exposure chambers 4 h per day for 15 days	C57BL/6 mice to study the effect of uranium mining dusts effect	*let-7a-5p, miR-143-3p, miR-151-5p, miR-28a-3p, miR-322-3p, miR-378c, miR-425-5p, miR-7a-5p, miR-874-5p*	RAF/MAP kinases, signaling by tyrosine kinases, NTRK (neurotrophin receptors), axon guidance and CRMPs (collapsin response mediator protein) in Sema3A signaling	Sanchez et al. (2020)
Regulation of neural proliferation and differentiation	PM_2.5_ > 200 μg/m^3^ via whole body system	Pregnant Sprague Dawley rats to study the effect of particulate on embryos neuronal development	*miR-214-3p, miR-6334, miR-1839, miR-149-5p, miR-667, miR-3548, miR-139-5p, miR-6324, miR-541-5p, miR-26b, miR-449c, miR-6332, miR-134-3p, miR-3065-3p, miR-105, miR-133c, miR-448-5p, miR-3072, miR-7a-2-3p*	Brain and somite development	Li et al. (2019)

### 5.6 The lung-brain axis miRNAs altered by PM exposure

As is now known in recent literature, the exposome conditions the genetic makeup, including miRNAs, inducing response processes, primarily inflammation and oxidative stress, which, in a cascade, modulate gene expression. Therefore, at the end of our bibliographic analyses regarding both the miRNAs associated to the lung-brain axis, and the miRNAs linked to air pollution exposure, we can conclude by crossing the two article databases that just 6 miRNAs resulted affected by PM exposure and being involved in lung-brain axis regulation: *miR-21, miR-143, miR-322, miR-149, miR-200, and miR-301*. Therefore, we speculated that those miRNAs should be deeply investigated by further analysis, by *in vivo* techniques in mice models. MiR-21 represents a crucial miRNA involved in the regulation of EMT, migration, and neuroinflammation by regulating ERK and STAT3 pathways (Tiong et al., 2023). Both miR-143 and miR-322 have been described as important regulators of the immune response and the permeability of the BBB (Sanchez et al., 2020). These miRNAs were observed to drive the expression of HIF1a, VEGF, and RAF/MAP kinase pathways. The last three miRNAs, miR-149, miR-200, and miR-301, have been described as significant regulators of cell proliferative activity (Cavallari et al., 2021; Fotakopoulos et al., 2023; Xue et al., 2021). They regulate both neuronal or astrocytic differentiation and proliferation rather than the proliferation of glioma cells or regulating lung fibrosis. Those miRNAs regulate PKM2 and BCYRN1 pathway (miR-149) (Lang et al., 2020), the TGFβ or STAT3/IL6 pathway (miR-301), and the EpCAM or SOX2 signaling pathways (miR-200c) (Zhao et al., 2018).

### 5.7 lncRNAs controlling lung brain axis linked to PM exposure

LncRNAs modulate miRNAs, playing crucial roles in cancer progression. They often regulate genes involved in tumor suppression and immune defense. Using a similar approach to identifying lung-brain axis miRNAs responsive to PM exposure, we selected seven lncRNAs: Metastasis Associated Lung Adenocarcinoma Transcript 1 (MALAT1), Brain Cytoplasmic RNA 1 (BCYRN1), Long Intergenic Non-Coding RNA 922 (LINC00922), linc-ZNF469-3, lnc-MMP2-2, FLVCR1-AS1 and lncTCF7.

MALAT1 is linked to cancer, poor prognosis, and brain/lung injuries. It modulates oxidative stress by regulating miR-140-5p, enhancing Nrf2 activity and reducing ROS (Qin and Xu, 2024). In cerebral ischemia-reperfusion injury, MALAT1 controls the miR-142-3p/SIRT1 axis, reducing TNF-α, IL-6, IL-1β, and increasing detoxifying enzymes like SOD and Catalase (Meng et al., 2023).

In Subarachnoid Hemorrhage (SAH) models, MALAT1 overexpression increases apoptosis and oxidative stress in neurons, possibly via miR-499-5p (Zhou et al., 2022). It also activates microglia, promoting inflammation, and is upregulated in glioma stem cells and their extracellular vesicles, which drive IL-6, IL-8, and TNF-α secretion (Yang et al., 2020). BCYRN1 is overexpressed in NSCLC, promoting cell motility and lymph node metastasis. Its silencing reduces A549 lung cancer growth. It enhances glycolysis, proliferation, and invasion via miR-149/PKM2 regulation (Song et al., 2022; Lang et al., 2020). Linc-ZNF469-3 is upregulated in lung-metastatic triple-negative breast cancer (TNBC), increasing EMT markers and metastasis by interacting with miR-574-5p, which suppresses ZEB1 (Wang et al., 2018).

As already reported, in some tumors lncRNAs exert their function by being transported by EVs and therefore influencing the behavior and responses of the tumor microenvironment. For instance, LINC00482 expression was found in the serum of NSCLC patients. In this case, it is associated with miR-142-3p expression, which in turn controls the expression of TGFb1 (Xu et al., 2023). In addition, the lnc-MMP2-2 served as a miRNA sponge or a competing endogenous RNA for miR-1207-5p and consequently modulated the repression of EPB41L5. In conclusion, TGF-β1-mediated exosomal lnc-MMP2-2 increases BBB permeability to promote NSCLC brain metastasis. Thus, exosomal lnc-MMP2-2 may be a potential biomarker and therapeutic target against lung cancer brain metastasis (Wu et al., 2021). Moreover, the rarely investigated lncRNA FLVCR1-AS1 is involved in various human cancers, including glioma where it is highly expressed and regulates miR-4731-5p upregulating E2F2 expression (Yan et al., 2020).

lncTCF7 is highly expressed in glioma tissues and cell lines which encourages the proliferation and migration of those cells, whereas its downregulation of lncTCF7 significantly suppresses the tumorigenesis of glioma. Mechanistically, lncTCF7 enhances the self-renewal of glioma cells by increasing the expression of epithelial cell adhesion molecules (EpCAM). The detailed molecular mechanism revealed that lncTCF7 binds to miR-200c and reduces the amount of miR-200c, which consequently weakens the negative regulation of miR-200c on EpCAM (Wang et al., 2018) (Table 4).

**TABLE 4:** LncRNA controlling lung brain axis.

lncRNA	miRNA	Axis or target	Function		Tissue	Ref.
MALAT1	*miR-140-5p*	MALAT1/miR-140-5p/Nrf2	Oxidative stress	Ischemia/reperfusion	Rat model, neuronal injury model	Qin and Xu (2024)
	*miR-142-3p*	miR-142-3p/SIRT1 axis		Cell proliferation	Mouse brain tissue cerebral ischemia-reperfusion injury	Meng et al. (2023)
	*miR-499-5p*	miR-499-5p/SOX6 axis		Apoptosis	Mouse model of subarachnoid hemorrhage	Zhou et al. (2022)
	*miR-129-5p*	miR-129-5p/HMGB-1 Axis	inflammatory response		Glioma stem cell	Yang et al. (2020)
BCYRN1	*miR-30b-3p*	miR-30b-3p/ROCK1 axis	Metastasis		NSCLC	Song et al. (2022)
	*miR-149*	miR-149/PKM2 axis	Glycolysis cell proliferation and invasion			Song et al. (2022)
linc-ZNF469-3	*miR-574-5p*	miR-574-5p-ZEB1 axis	Metastasis		Triple-negative breast cancer	Wang et al. (2018)
LINC00482	*miR-142-3p*	miR-142-3p/TGF-β1 axis	Metastasis		NSCLC	Xu et al. (2023)
lnc-MMP2-2	*miR-1207-5p*	miR-1207-5p/EPB41L5 axis	Migration, BBB permeability		Endothelial monolayers and mouse models	Wu et al. (2021)
FLVCR1-AS1	*miR-4731-5p*	LVCR1-AS1/miR-4731-5p/E2F2 axis	Metastasis		Glioma tissues and cell lines	Yan et al. (2020)
lncRNATCF7	*miR-200c*	miR-200c-EpCAM axis	Tumorigenesis		Glioma tissues and cell lines	Zhao et al. (2018)

## 6 Conclusion

Nowadays ambient air pollution is a worldwide public health emergency. Despite many studies on air pollutant-associated health effects, the underlying molecular mechanisms of non-communicable disease (i.e., neurological, oncological, and non-hereditary disease) remain mainly unclear.

Toxicological studies showed that exposure to PM is directly associated with impairment of the respiratory, intestinal, cardiovascular, and nervous systems functions. Animal models can be tailored to specific research questions, such as investigating disease mechanisms or susceptibility to certain conditions. To study the health adverse effects induced by PM exposure, several models have been discussed, both for chronic, sub-chronic, or acute exposure.

PET imaging represents a powerful tool for non-invasively monitoring the *in vivo* PM-induced alterations in the lung-brain axis (tissue inflammation; tumor proliferation and invasion; amyloid deposition; neurotoxicity, neuroinflammation) using specific radiotracers. By elucidating the mechanisms underlying these effects, PET imaging could contribute to the understanding of air pollution-related health risks and facilitate the development of targeted interventions.

Considering that air pollutant exposure has the potential to change the miRNA profiles, and that miRNAs have a main role in the control of non-communicable diseases (NCDs), such as neurodegenerative and oncological diseases, miRNA deregulation may be considered as an early marker of PM effect on human health. In particular, by a metanalytic approach, we found 6 miRNAs (*miR-21, miR-143, miR-322, miR-149, miR-200*, and *miR-301*) involved in the lung-brain axis being linked to PM exposure. Some of these miRNAs could be also controlled by specific lncRNA (i.e., LINC00482 for *miR-21*), but none of the seven PM-respondent lncRNAs (MALAT1, BCYRN1, LINC00922, linc-ZNF469-3, lnc-MMP2-2, FLVCR1-AS1, and lncTCF7) selected by the metanalytic approach is a known regulator of this group of miRNAs; this result suggests that other miRNA could emerge as PM responders in the lung-brain axis. The six miRNAs associated with the lung-brain axis affected by PM could have a main role in the regulation of BBB integrity and functions, neuronal cell proliferation and differentiation, and neuroinflammation. Further studies are needed to elucidate this network in NCDs.
