# Supplementary figures and images for: Effects of particulate air pollution exposure on lung-brain axis and related miRNAs modulation in mouse models
Source: Front Cell Dev Biol. 2025 Mar 20;13:1526424. doi: 10.3389/fcell.2025.1526424 (PMC12003928; doi:10.3389/fcell.2025.1526424)

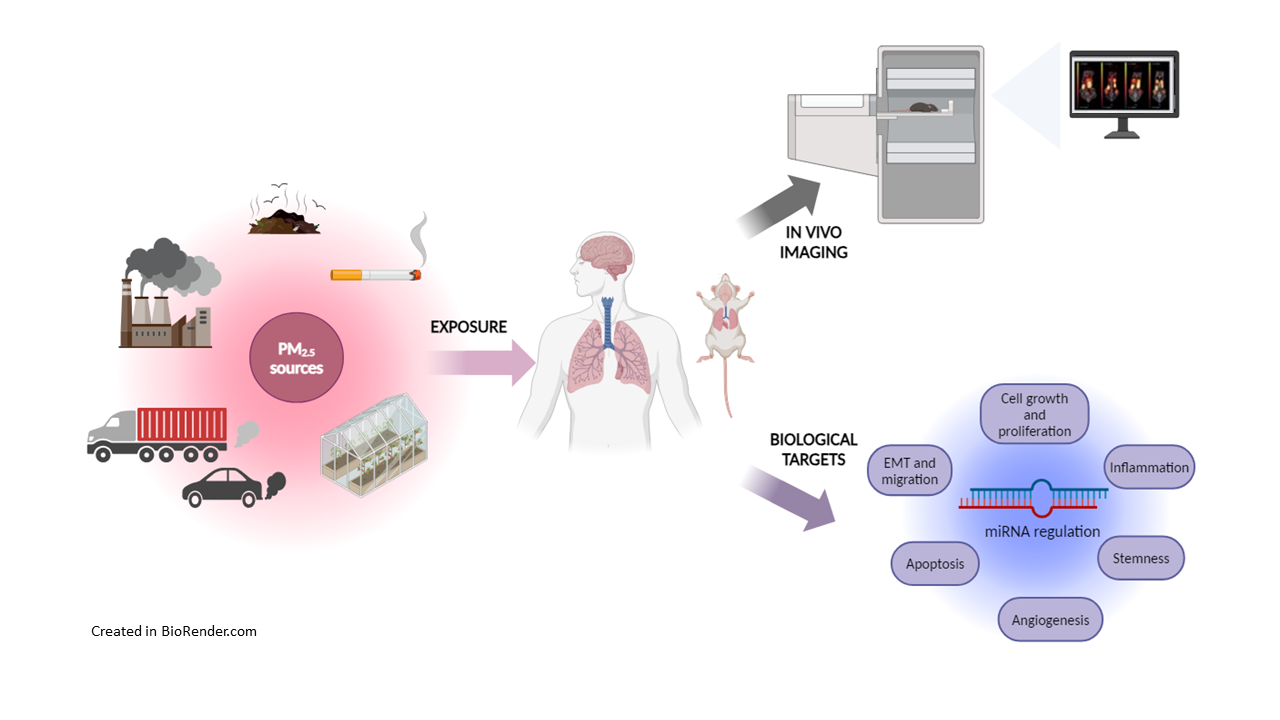

Supplement: Supplementary file 1 [file Image1.tif]
